# *Anaplasma phagocytophilum*, *Babesia microti*, and *Borrelia burgdorferi* in *Ixodes scapularis*, Southern Coastal Maine

**DOI:** 10.3201/eid1004.030566

**Published:** 2004-04

**Authors:** Mary S. Holman, Diane A. Caporale, John Goldberg, Eleanor Lacombe, Charles Lubelczyk, Peter W. Rand, Robert P. Smith

**Affiliations:** * Vector-borne Disease Laboratory, Maine Medical Center Research Institute, Portland, Maine, USA; † University of Maine, Orono, Maine, USA

**Keywords:** Ixodes scapularis, Lyme disease, Borrelia burgdorferi, Anaplasma phagocytophilum, Babesia microti

## Abstract

*Ixodes scapularis* (deer ticks) from Maine were tested for multiple infections by polymerase chain reaction and immunofluorescence. In 1995, 29.5%, 9.5%, and 1.9% of deer ticks were infected with *Borrelia* *burgdorferi*, *Anaplasma phagocytophilum*, and *Babesia* *microti*, respectively. In 1996 and 1997, the number of *A. phagocytophilum-*infected ticks markedly declined. In 1995 through 1996, 4 (1.3%) of 301 were co-infected.

Throughout its range in the eastern and upper midwestern United States, *Ixodes scapularis* (*Ixodes dammini*) (deer tick) is the vector of *Borrelia burgdorferi*, the causative agent of Lyme disease. In recent decades, it has been associated with several other pathogens, including bacteria, viruses, and protozoa, a guild of pathogens similar to that seen in the related tick *Ixodes ricinus* in Europe (*1*).

*I. scapularis* was determined to be the vector of the intraerythrocytic protozoan *Babesia microti* on Nantucket Island, Massachusetts in 1979 (*2*). Human granulocytic ehrlichiosis (HGE) was first described in 1994 in patients from Wisconsin and Minnesota (*3*). *I. scapularis* was determined to be a competent vector of the obligate intracellular bacteria that cause HGE, and field-derived ticks from Massachusetts were found to be co-infected with the HGE agent and *B. burgdorferi* (*4*). The agent of HGE, previously referred to as *Ehrlichia phagocytophila*, has recently been reclassified as *Anaplasma phagocytophilum* (*5*).

Rodents and birds have been demonstrated to be reservoirs of the Lyme disease spirochete in areas of Maine where the tick is established (*6*). This study sought to determine if *I. scapularis* at the northern edge of its range was infected with *A. phagocytophilum* and *Ba. microti*, in addition to *B. burgdorferi.*

## The Study

*I. scapularis* nymphs and adult females that had partially fed on a variety of hosts were collected in 1995 through 1997 from coastal areas in Maine, from York to Hancock counties, where the tick is established and Lyme disease is endemic (Figure A). Ticks removed from pets and humans were submitted to our laboratory for species confirmation. Nymphs were also removed from white-footed mice and eastern chipmunks live-trapped on established research grids in the town of Wells and from Norway rats trapped on an offshore island. Mammal trapping procedures were approved by the Maine Medical Center Institutional Animal Care and Use Committee. One *I. scapularis* female was removed from a nontranquilized, live, white-tailed deer that had become accustomed to humans on Monhegan Island. All ticks were transported alive to the laboratory.

**Figure:**
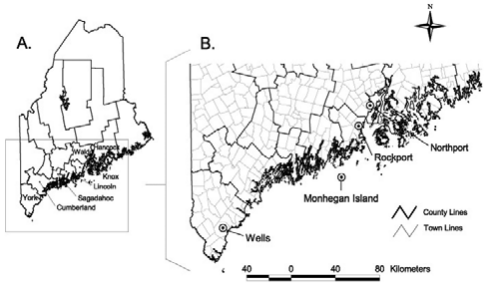
A. Counties in southern Maine where ticks were collected. B. Four towns where ticks infected with *Anaplasma phagocytophilum* or *Babesia microti* were found.

Ticks were dissected on sterile glass slides in a drop of 10 mmol Tris-HCl, 1 mmol EDTA pH 8 (TE). Salivary glands were isolated, and one gland from each tick was stained by the Feulgen reaction for microscopic examination for inclusions (*7*); the other gland was prepared for DNA extraction. A smear of tick midgut was prepared for fluorescent microscopic examination for spirochetes as described previously (*6*).

All polymerase chain reaction (PCR) tests were performed on salivary glands from individual ticks except for 14 instances in 1995 when salivary glands from several ticks collected from an individual host were pooled for PCR analysis. For statistical purposes, when a PCR product was obtained from a pool of salivary glands from multiple ticks, only one tick in the pool was assumed to be infected.

Salivary glands were stored at –20°C in 50 μL of TE buffer until DNA extraction. DNA was isolated using a standard phenol/chloroform extraction procedure (*8*) or by using the IsoQuick kit (ORCA Research, Bothell, WA) according to the manufacturer’s protocol and placed in 20 μL of TE buffer. Sterile aerosol-barrier tips were used during all procedures. DNA isolation and PCR reactions were performed in separate laboratories. Positive and negative controls were included in each PCR reaction.

*Babesia* was detected by amplifying a 437-bp portion of the eukaryotic 18S rRNA gene by PCR using primer pair PiroA/PiroB (*9*). Components were denatured at 94°C for 45 sec, annealed at 60°C for 45 sec, and extended at 72°C for 2 min, for a total of 40 cycles. Samples were separated by electrophoresis on a 1% Sea Plaque agarose gel containing ethidium bromide and 40 mmol Tris-acetate 1 mmol EDTA pH 8.3 buffer.

*Anaplasma* was identified by the amplification of 16S rDNA by PCR. The primer pair consisting of GE9 (*3*) and Ehr747 (*10*) was used to generate an 849-bp fragment. The thermal cycling profile used was the same as for *Babesia*.

Amplified products were excised from the gels, treated with Beta-agarase (Sigma, St. Louis, MO), cycle-sequenced using dye-labeled dideoxy terminators (Applied Biosystems Big Dye Reaction Kit, Foster City, CA) and purified by using Centri-Sep columns (Princeton Separations, Adelphia, NJ). Samples were electrophoresed on a 6% polyacrylamide stretch gel using an ABI 373A DNA sequencer. DNA sequences were compared with previously published sequences for species identification, using the Sequence Navigator program by Applied Biosystems.

From 1995 to 1997, PCR was performed on salivary glands from 223 *I. scapularis* nymphs and 171 females. Nymphs made up 44% of ticks tested the first year of the study and 61% in both of the later years. Table presents the prevalence of infection with *A. phagocytophilum*, *Ba. microti*, and *B. burgdorferi* in *I. scapularis* studied each year.

Four of the positive PCR results were obtained from pooled glands. Assuming only one gland in each pool was positive, a total of six nymphs (possible range 6–12) and five female *I. scapularis* (possible range 5–7) were infected with *A. phagocytophilum*. *Ba. microti* was found in two nymphs and one female tick. Nine of the infected ticks were collected in the town of Wells in York County, three were from Monhegan Island in Lincoln County, and one each was from the towns of Rockport in Knox County and Northport in Waldo County (Figure B). Four nymphs were infected with two organisms (Table). All of the co-infected ticks were from the town of Wells in York County.

**Table:** Prevalence of *Anaplasma phagocytophilum*, *Babesia microti*, and *Borrelia burgdorferi* in *Ixodes scapularis*, Maine

Y	n	No. (%)					
		*A. phagocytophilum* No. (%)	*Ba. microti*	*B. burgdorferi*	*B. burgdorferi* *and* *A. phagocytophilum*	*B. burgdorferi* *and* *Ba. microti*	
1995	105	10 (9.5)^a,b^	2 (1.9)^a^	31 (29.5)^a^	2 (1.9)	1 (1.0)	
1996	196	1 (0.5)	1 (0.5)^a^	35 (17.9)^a^	0	1 (0.5)	
1997	93	0	0	22 (23.7)	0	0	

^a^Total includes co-infected ticks. ^b^Four pools of salivary glands from 2–3 ticks from the same host tested positive. This table presents data assuming only one tick from each pool was infected.

*Babesia* spp. piroplasms were microscopically visualized by the Feulgen reaction in salivary acini from 21 ticks. Two glands positive for *Babesia* spp. by visual inspection had PCR product that matched sequences for *Ba. microti*; the remaining 19 (90.5%) of 21 samples matched sequences for *Ba. odocoilei*, a parasite of deer not known to cause human illness (*9*). Two (18%) of 11 feulgen-stained glands from ticks determined to be positive for *A. phagocytophilum* by PCR were considered positive by visual inspection of the other gland. All amplification product from the *A. phagocytophilum*–positive ticks had 99% homology (848/849 bp) with sequences of *E. phagocytophila*-human agent of Chen et al. (GenBank accession no. U02521) (*3*).

## Conclusions

Multiple studies conducted in hyperendemic areas of Lyme disease have reported *A. phagocytophilum* and *Ba. microti* in field-collected *I. scapularis* (*4*,*7*,*10*–*13*). Schwartz et al. reported an increase in the percent of adult deer ticks infected with the agent of HGE in Westchester County, New York from 32% of ticks collected in1984 and tested retrospectively, to 53% in 1995 (*11*). In a 2-year study in Connecticut, 12.5% of adult ticks in 1996 and 19% in 1997 were infected with *A. phagocytophilum* (*12*). The current study showed a decrease in the percent of infected ticks collected from the same geographic areas for a 3-year period. *A. phagocytophilum* infection rates declined from 9.5% in 1995 to 0.5% and 0% in subsequent years. The percent of ticks infected with *B. burgdorferi* remained relatively constant for the 3-year period (Table).

*Ba. microti* infection rates based on DNA sequences of the organism have been reported from 5% of adult ticks tested in New Jersey (*13*) to 9% of adult ticks on Nantucket Island in Massachusetts (*4*). In 1995, 1.9% of ticks tested in this study were positive for *Ba. microti*; the percent infected dropped in subsequent years to 0.5% and 0%. This low prevalence of *Ba. microti* infection in Maine ticks is not unexpected. Mather et al. reported that *Ba. microti* was found only in areas of Rhode Island where tick abundance reached >20 nymphs per hour of flagging (*14*). In our study, the three ticks infected with *Ba. microti* were collected in the town of Wells in coastal York County where tick density is the highest in the state (unpub. data). Although enzootic *Ba. microti* maintained by *Ixodes angustus* or other nidicolous ticks may be widespread in Maine, *I. scapularis* density high enough to support zoonotic transmission of *Ba. microti* may only occur in a few foci (*15*).

That the prevalence of infection of ticks with *B. burgdorferi* during this 3-year study remained fairly constant while that of *A. phagocytophilum* showed greater variation is of interest. Other researchers have shown that white-footed mice remain reservoir competent for *A. phagocytophilum* for short periods of time (*16*) and that transmission of multiple organisms may have a different dynamic than that of single pathogens (*17*). Few studies have followed the natural infection of tick hosts with multiple organisms over time. This study indicates that the prevalence of these emerging pathogens may not be as stable from year to year as is the rodent-*I. scapularis*-*B. burgdorferi* cycle.

This study provides evidence of the potential for human exposure to multiple tick-borne pathogens in southern coastal Maine and that the risk for exposure to *A. phagocytophilum* may vary considerably from year to year.
